# Dosing optimization of rituximab for primary membranous nephropathy by population pharmacokinetic and pharmacodynamic study

**DOI:** 10.3389/fphar.2024.1197651

**Published:** 2024-03-26

**Authors:** Hao Liang, Zhenling Deng, Shu Niu, Weijie Kong, Yang Liu, Song Wang, Haiyan Li, Yue Wang, Danxia Zheng, Dongyang Liu

**Affiliations:** ^1^ Department of Nephrology, Peking University Third Hospital, Beijing, China; ^2^ Drug Clinical Trial Center, Peking University Third Hospital, Beijing, China; ^3^ Department of Pharmacology, College of Pharmacy, Inner Mongolia Medical University, Hohhot, China; ^4^ Institute of Medical Innovation, Peking University Third Hospital, Beijing, China; ^5^ Beijing Key Laboratory of Cardiovascular Receptors Research, Peking University Third Hospital, Beijing, China

**Keywords:** rituximab, primary membranous nephropathy, population pharmacokinetic and pharmacodynamic, target-mediated drug disposition, dosage optimization

## Abstract

Primary membranous nephropathy (PMN) is the most common cause for adult nephrotic syndrome. Rituximab has demonstrated promising clinical efficacy by random controlled trials and the off-label use is widely adopted in PMN. However, the standard dosage is borrowed from B cell lymphoma treatment with far more antigens and is oversaturated for PMN treatment, accompanied with additional safety risk and unnecessary medical cost. More than 15% serious adverse events were observed under standard dosage and low dose therapies were explored recently. Dose optimization by clinical trials is extremely time- and cost-consuming and can be significantly accelerated with the aid of model-informed drug development. Here, we aim to establish the first population pharmacokinetic and pharmacodynamic (PPK/PD) model for rituximab in PMN to guide its dosage optimization. Rituximab pharmacokinetic and pharmacodynamic data from 41 PMN patients in a retrospective study under a newly proposed monthly mini-dose were used to construct quantitative dose-exposure-response relationship via mechanistic target-mediated drug disposition (TMDD) model followed by regression between the reduction of anti-PLA2R titer and time after the treatment. The final model, validated by goodness-of-fit plots, visual predictive checks and bootstrap, was used to recommend the optimized dosing regimen by simulations. The model was well validated for PK/PD prediction. The systemic clearance and half-life are 0.54 L/h and 14.7 days, respectively. Simulation of a novel regimen (6 monthly doses of 100 mg) indicated the comparable ability and superior duration time of CD20^+^ B cell depletion compared with standard dosage, while the cumulative dosage and safety risk was significantly decreased. We established the first PPK/PD model and provide evidence to support the dosage optimization based on monthly mini-dose. Our study can also efficiently accelerate dosage optimization of novel anti-CD20 antibodies in PMN and other indications.

## 1 Introduction

Membranous nephropathy (MN) is the one of the most predominate causes of adult nephrotic syndrome (NS). About 80% of MN patients are primary MN (PMN) with no underlying cause, while one-third PMN patients progress to advanced kidney failure (Ronco et al., 2021). Podocyte phospholipase A2 receptor (PLA2R) was identified as the major antigen in PMN responsible for the formation of immune complex deposition on basement membranes that induces podocyte injury and proteinuria (Beck et al., 2009). The pathophysiological role of PLA2R provides a clear rational that the decrease of anti-PLA2R titer precedes the remission of proteinuria (Ruggenenti et al., 2015). Thus, PMN remission can be predicted by anti-PLA2R antibody reduction whereas relapse occurred with antibody re-emergence.

As an autoimmune disease, immunosuppressive therapies are widely used to treat PMN. However, traditional immunosuppressive treatments have long been suffered from high incidence of adverse effect and relapse rate. Rituximab (RTX), a chimeric monoclonal anti-CD20 antibody, eliminates CD20^+^ B cells through antibody-mediated cytotoxicity (ADCC) and complement-mediated cytotoxicity (CDC) effect (Boross and Leusen, 2012). RTX was primarily approved for the treatment of B cell lymphoma and then further extended to autoimmune disease. RTX offers a selective B cell targeting approach that prevent antibody production, and thus was introduced into PMN treatment (Remuzzi et al., 2002). Random controlled trials demonstrated that RTX was noninferior to cyclosporine in terms of 12-month proteinuria remission and was superior in the duration of proteinuria remission up to 24 months (Dahan et al., 2017; Fervenza et al., 2019). Accordingly, RTX has been suggested as first-line treatment for PMN by the latest KIDGO guideline (Rovin et al., 2021).

Although the off-label use of RTX was widely adopted for PMN treatment, the optimal dosage is still in debate. The dosage was borrowed from B cell lymphoma treatment and recommended as four weekly infusions of 375 mg/m^2^ or two 1000 mg infusions with 2 weeks apart (Rovin et al., 2021). However, the B cell counts in PMN patients are only 1/300 of that in lymphoma patients., Thus, the standard dose may be oversaturated for B cell depletion in PMN patients even with the urinary loss of rituximab and cause unnecessary medical cost (Bensalem et al., 2022), though the safety risk is relatively low when compared with other immunosuppressive drugs (Fervenza et al., 2019). It is still debatable for the relationship between larger doses and improved clinical outcomes. For example, studies by Gabriella Moroni et al. and Roberta Fenoglio et al. indicated that the efficacy is similar under low dose or high dose of rituximab (Moroni et al., 2017; Fenoglio et al., 2021). Although some studies demonstrated that high dose therapy yield higher remission rate (Seitz-Polski et al., 2019), further analysis indicated that the remission is associated with residual rituximab levels at month-3 (Boyer-Suavet et al., 2019), which can serve as an effective predictor for remission (Teisseyre et al., 2021). Accordingly, it is more effective to maintain the residual rituximab level for a longer time than to have high peak concentration. A retrospective study was carried out by our group and revealed that monthly mini-dose (100 mg) has better short-term (6 months) clinical efficacy compared to the standard dose regimens while the cumulative dose and the rate of adverse event is much lower (Wang et al., 2023). Inspired by the promising preliminary results, further studies are urgently warranted to compare with standard dosage and better optimize the dosage.

It is very time- and cost-consuming to explore the optimal dosage by conducting clinical trials, especially for PMN that requires long-term (up to 24 months) observations. Model-informed drug development strategy is a powerful tool to aid and accelerate dose optimization by constructing the quantitative dose-exposure-response relationship and accurately predicting the clinical efficacy of different regimens in advance (Cardone et al., 2011). However, the pharmacokinetic (PK) characteristics were rarely described for RTX in PMN treatment and the pharmacokinetic and pharmacodynamic (PK/PD) relationship of RTX has not been established yet (Bensalem et al., 2022). Although CD20^+^ B cell depletion is not the clinical endpoint for the treatment of PMN, it can serve as an efficient surrogate that bridge the PK of RTX and clinical efficacy. Here, we constructed the first population PK/PD (PPK/PD) model that revealed the quantitative relationship between dosage, RTX concentration, CD20^+^ B cell count and anti-PLA2R titer, and enabled us to effectively optimize dosage according to simulation results. Our study provides concrete evidence to find the optimal dose with significantly reduced SAE occurrence and medical burden for PMN patients on the basis of monthly mini-dose.

## 2 Materials and methods

### 2.1 Patient population and study design

In our retrospective study, a total of 41 PMN patients who received RTX treatment from March 2019 to December 2021 at Department of Nephrology, Peking University Third Hospital were included. Most patients received monthly mini-dose of 100 mg RTX, whereas some of them received 200–500 mg RTX for a few months with the intention to better control disease progress. Due to COVID-19 epidemic, part of patient population experienced a gap up to 10 months and received RTX infusion again afterwards. The trial was consistent with the Good Clinical Practice (GCP) standards and was registered at Chinese clinical trial registry (ChiCTR2200057381).

### 2.2 PK and PD data

The trough and peak RTX blood samples were collected before and after RTX infusion, respectively. RTX concentrations were detected by ELISA kit purchased from Abcam (Cambridge, MA, United States). RTX PK observations below the limit of quantification (3 ng/mL) were excluded from the analysis. CD20^+^ B cell count was measured before every RTX intravenous (IV) infusion. CD20^+^ B cell depletion was defined as an absolute count < 5/μL in peripheral blood (Cravedi et al., 2007). Serum anti-PLA2R titer was assessed by an ELISA kit purchased from Euroimmune, Lubeck, Germany.

### 2.3 PPK/PD model development

PPK/PD models were constructed by nonlinear mixed-effects modeling methods to describe the PK characteristics and variability for RTX concentration and CD20^+^ B cell count (Supplementary Figure S1). The observed RTX concentrations were converted to molar units. The CD20^+^ B cell counts were transformed to CD20 concentration in molar units by multiplying 94000 CD20 molecules per cell and dividing by Avogadro’s constant (Ginaldi et al., 1998).

A total of four structural PPK models were developed as follows. At first, a basic two-compartment model was constructed as reported by many previous studies that is characterized by linear elimination (Ng et al., 2005; Regazzi et al., 2005; Li et al., 2007; Blasco et al., 2009). Based on the basic two-compartment model, two additional models were constructed with nonlinear elimination approximation described by Michaelis-Menten elimination (Tout et al., 2017) and time-varying elimination (Li et al., 2012), respectively. Finally, a mechanistic target-mediated drug disposition (TMDD) model with quasi-steady-state (QSS) approximation was constructed. In this model, the free RTX in central compartment is directly eliminated by a first-order elimination rate constant (*k*_*el*_). In addition, central compartment RTX distributes to and from peripheral compartment by first-order distribution rate constant *k*_*pt*_ and *k*_*tp*_, respectively. In plasma, RTX binds to CD20 antigen (R) with a second-order rate constant (*k*_*on*_) to form drug-receptor complex (RC), whereas RC dissociates at a first-order rate (*k*_*off*_) and internalizes for degradation (*k*_*int*_). CD20 is reported to be a non-internalizing receptor, and the elimination of CD20 and the subsequent depletion of CD20^+^ B cells is thought to be via ADCC and CDC effect (Boross and Leusen, 2012). Thus, the internalization rate constant *k*_*int*_ was replaced by the RTX-CD20 complex target-mediated elimination rate constant (*k*_*tmd*_), which is assumed to be the maximal rate of elimination observed for mAbs in clinical use (Glassman and Balthasar, 2017). On the other hand, CD20^+^ B cell is synthesized at a zero-order rate (*k*_*syn*_) and degraded at a first-order rate (*k*_*deg*_) in the absence of RTX binding. Due to its non-internalization character, the turnover rate of CD20 was assumed to be similar to the disappearance rate of B cells from circulation (∼3.9% per day) (Macallan et al., 2005). The RTX-CD20 complex elimination rate constant (*k*_*tmd*_) is much greater than the dissociation rate constant *k*_*off*_ (Bondza et al., 2017). Thus, QSS approximation of TMDD model was used, where QSS constant *k*_*ss*_ is defined as the sum of dissociation constant *k*_*D*_ and the quotient of *k*_*tmd*_ and dissociation rate constant (*k*_*off*_) (Gibiansky et al., 2008). Models were evaluated with objective function value (OFV), while the model with the lowest OFV and better fitting of the diagnostic plots was selected as structural model.

Between-subject variability (BSV) was modeled in an exponential form (Eq. 1):

$$P_{i}=TVP\times e^{\eta_{i}}$$ (1)

where $P_{i}$ is the *i*th individual value of the parameter; TVP is the typical value of the population parameter; η_*i*_ is the empirical Bayes estimates of BSV for the *i*th individual that is normally distributed with mean zero and variance ω^2^.

The residual variabilities were assessed as an error model in a proportional form (Eq. 2):

$$C_{ij}=\overset{\wedge }{C_{ij}}\times \left(1+\varepsilon_{pro}\right)$$ (2)

where $C_{ij}$ and $\overset{\wedge }{C_{ij}}$ is the *i*th observed and predicted concentration for the *j*th individual, respectively; $\varepsilon_{pro}$ is the proportional error component that is normally distributed with mean zero and variance σ^2^.

### 2.4 Covariate model development

Covariates with high correlation (correlation coefficient > 0.3) were taken into consideration for further the covariate analysis, including age, sex, eGFR, etc. Continuous and categorical covariates were established by a power function and a linear function (Eqs 3, 4), respectively:

$$P_{i}=\theta_{1}\times {\left(\frac{Cov_{con}}{Cov_{median}}\right)}^{\theta_{2}}$$ (3)

$$P_{i}=\theta_{1}\times \left(1+\theta_{2}\times Cov_{cat}\right)$$ (4)

Where P_*i*_ is the parameter for the *i*th individual; $Cov_{con}$ and $Cov_{cat}$ is the value of continuous and categorical covariate for the *i*th individual, respectively; $\theta_{1}$ is the typical value for the population parameter; $\theta_{2}$ is the estimated value for the covariate effect.

If two or more covariates were highly correlated, only the most clinically and practically relevant covariate was tested on the PK parameter in the analysis. These covariates were further screened using the SCM implemented in PsN.

### 2.5 Model evaluation

Goodness-of-fit (GOF) plots were used to evaluate the final model, which defined as the combination of four plots including the observed concentration (DV) *versus* the population predicted concentration (PRED) or individual predicted concentrations (IPRED) and conditional weighted residuals (CWRES) *versus* PRED or time. The predictive performance of the final model was evaluated using visual predictive checks (VPCs), which were repeated 1000 times to compare the DV data and simulated value with the 95% confidence intervals for the 5^th^,50th and 95th percentiles.

The results of bootstraps with 500 replicates were finally used to assess the final model. The RSEs of the parameter estimates and the condition number of the model were also considered.

### 2.6 Simulations

Using the established model, we carried out simulations for both standard dosage and monthly mini-dose to predicted RTX concentrations and CD20^+^ B cell counts under different regimen groups. In addition, mini-dose with larger infusion interval was also simulated.

### 2.7 Exponentially decrease function of anti-PLA2R titer

To explore the relationship between CD20^+^ B cell counts and anti-PLA2R titer, the individual anti-PLA2R titer data at descending stage was extracted, while serology relapse data that was defined as two consecutive ascending anti-PLA2R titer data was excluded. Anti-PLA2R titers detected with interval longer than 3 months or after 12 months since the first RTX infusion were excluded as well. The anti-PLA2R titer data was plotted *versus* time and described using an exponentially decrease function (Eq. 5). For patients experienced relapse and second round of anti-PLA2R reduction, two exponentially decrease functions using respective data were used.

$$anti-PLA2R\;titer=A\times e^{-k_{e,PLA2R}\times t}$$ (5)

### 2.8 Non-compartmental analysis

Non-compartmental analysis was conducted for the simulated results using typical PK parameters. The area under the concentration-time curve (AUC), the elimination half-life (t_1/2_), the apparent clearance (CL/F) and apparent volume of distribution during the terminal phase Vz (Vz/F) were determined.

### 2.9 Software

All the modeling and simulation were carried out with the first order conditional estimation method with interaction that is implemented within NONMEM 7.2 and aided by PsN. The dataset was treated and processed by R package. Pirana was used for documentation of the development process. Non-compartmental analysis was conducted using Phoenix WinNonlin V8.3.3 (Certara, Princeton, NJ, United States).

## 3 Results

### 3.1 Demographic characteristics and clinical response

A summary of subject demographic characteristics is shown (Table 1). A total of 31 male (75.6%) and 10 female (24.4%) patients were enrolled. The ages of the patients ranged from 19 to 76 years, with weight ranging from 50 to 101 kg (75.3 ± 11.3 kg). The albumin concentration and urine protein at baseline is 25.8 ± 5.7 g/L and 8.0 ± 3.6 g/day, respectively. The anti-PLA2R antibody titer ranged from 5.4–2695 U/mL (260.3 ± 453.2 U/mL), while 17 patients had a high anti-PLA2R antibody titer (>150 U/mL). The ratio of CD19^+^ B cell depletion at month 3 was 80% (33/41). The mean follow-up period was 15.9 (6–44) months. The cumulative dose of rituximab at month 6, month 12 and last follow-up was 578 ± 350, 923 ± 544 and 1060 ± 705 mg, respectively. The urinary protein levels decreased and serum albumin levels increased gradually after rituximab treatment, while the eGFR remained relatively stable. The remission rate was 21.2% (7/33) with 0% complete remission (CR) at 3 months, 50% (19/38) with 5.3% CR (2/38) at 6 months, 72.7% (24/33) with 18.2% CR (6/33) at 12 months, and 81.6% (30/38) with 28.9% (11/38) CR at last follow-up, respectively. Rate of relapse was 7.9% (3/38) at last follow-up.

**TABLE 1:** Demographic characteristics and clinical response of the study population at baseline and after treatment.

Variable (unit)	Overall cohort (*n* = 41)	Monthly 100 mg RTX (*n* = 31)	Monthly>100 mg RTX (*n* = 10)
Female Sex	10 (24.4)^a^	7 (22.6)	3 (30)
Age (years)	52.8 ± 14.9 (19–76)	54.5 ± 14.6 (19–76)	47.5 ± 15.3 (23–76)
Weight (kg)	75.4 ± 11.3 (50–101)	74.6 ± 10.6 (59–101)	77.2 ± 13.6 (50–95)
Baseline			
Serum albumin (g/L)	25.8 ± 5.7 (15.3–40.9)	25.0 ± 4.4 (15.3–36.3)	28.1 ± 6.9 (16.1–40.9)
Urine protein (g/day)	8.0 ± 3.6 (2–15.2)	8.3 ± 3.0 (4.2–15.2)	6.7 ± 4.5 (2–15)
eGFR (mL/min/1.73 m^2^)	92.0 ± 24.6 (32–151)	92.5 ± 23.4 (32–151)	90 ± 29.8 (36–128)
Anti-PLA2R antibody titer (U/mL)	260.3 ± 453.2 (5.4–2695)	304.7 ± 513.7 (5.4–2695)	122.6 ± 71.7 (44.2–255)
Patients with high anti-PLA2R titer (>150)	17 (41.5)	14 (45.2)	3 (30)
3 months			
Serum albumin (g/L)	28.4 ± 5.6 (18.1–40.2)	29.4 ± 6.0 (18.1–40.2)	26.0 ± 3.8 (20.0–32.1)
Urine protein (g/day)	7.4 ± 3.7 (1.1–14.7)	7.1 ± 3.6 (1.1–12.5)	8.3 ± 4.1 (1.3–14.7)
eGFR (mL/min/1.73 m^2^)	88.1 ± 32.0 (15–143)	88.0 ± 30.4 (34–143)	88.4 ± 37.9 (15–132)
CD19^+^ B cell depletion at month 3	33 (80)	25 (80.6)	8 (80)
PR + CR, n (%)^b^	7/33 (21.2)	6/23 (26.1)	1/10 (10)
CR, n (%)	0/33 (0)	0/23 (0)	0/10 (0)
RTX cumulative dose (mg)	405 ± 260	330 ± 95	610 ± 425
6 months			
Serum albumin (g/L)	31.3 ± 4.7 (22.9–40.6)	32.0 ± 5.2 (22.9–40.6)	29.8 ± 3.0 (26.3–35.1)
Urine protein (g/day)	4.5 ± 3.2 (0.1–12.3)	4.3 ± 3.1 (0.1–11.3)	5.1 ± 3.6 (1.1–12.3)
eGFR (mL/min/1.73 m^2^)	85.5 ± 28.4 (19–133)	86.7 ± 24.8 (33–132)	82.4 ± 37.6 (19–133)
PR + CR, n (%)	19/38 (50)	15/28 (53.6)	4/10 (40)
CR, n (%)	2/38 (5.3)	2/38 (5.3)	0/10 (0)
RTX cumulative dose (mg)	578 ± 350	460 ± 140	930 ± 533
12 months			
Serum albumin (g/L)	35.9 ± 6.4 (20.0–46.1)	36.1 ± 6.7 (20.0–46.1)	35.0 ± 4.5 (30.5–41.8)
Urine protein (g/day)	2.1 ± 2.2 (0.1–10.0)	2.1 ± 2.3 (0.1–10.0)	1.9 ± 1.4 (0.9–4.3)
eGFR (mL/min/1.73 m^2^)	84.9 ± 24.8 (38–131)	83.7 ± 24.8 (38–131)	91.0 ± 26.4 (53–119)
PR + CR, n (%)	24/33 (72.7)	19/27 (70.4)	5/6 (83.3)
CR, n (%)	6/33 (18.2)	6/27 (22.2)	0/6 (0)
RTX cumulative dose (mg)	923 ± 544	713 ± 319	1550 ± 608
Last follow-up			
PR + CR, n (%)	30/38 (81.6)	23/28 (82.1)	8/10 (80)
CR, n (%)	11/38 (28.9)	11/28 (39.3)	0/10 (0)
RTX cumulative dose (mg)	1060 ± 705	807 ± 525	1820 ± 639
Rate of relapse	3/38 (7.9)	2/28 (5.3)	1/10 (10)
Length of follow-up (months)	15.9 (6–44)	16.2 (6–44)	15.1 (6–38)

^a^ Values are displayed as median (range) or n (%).

^b^ Patients (*n* = 3) who were followed up for less than 3 months were not evaluated for clinical remission and relapse.

CR, complete remission; PR, partial remission; RTX, rituximab.

### 3.2 PK and PD data

A total of 171 RTX concentration, 220 CD20^+^ B cell count and 276 PLA2R titer data from 41 PMN patients were collected. Most patient receive multiple doses of 100 mg RTX infusion with at least 1 month apart, while part of them received 200–500 mg RTX infusion.

### 3.3 PPK/PD model construction

Four PPK models were constructed to describe RTX PK profile, including two-compartment model with linear, time-varying and Michaelis-Menten. An additional PPK/PD model described by TMDD model with QSS approximation was established. Among four models, TMDD model best captured the RTX concentration and CD20^+^ B cell count profile with lowest OFV (Supplementary Table S1), and was thus selected as structural model. The model has included both specific elimination (k_tmd_) and non-specific elimination (k_el_). The contribution of both proteolysis and urinary loss have been considered in our model, although it is difficult to exactly evaluate their respective contribution due to limited data.

### 3.4 Covariate model and final model

More than 30 baseline physiological parameters were used for covariates screening. Notably, covariates have been identified for RTX in many other diseases, such as body weight, sex, age on clearance and body surface area on central volume (Wang et al., 2020). However, no covariates were identified in our study. Thus, the final model was replaced by the base structural model and the results of estimated model parameters were presented (Table 2).

**TABLE 2:** Estimated PPK/PD final model parameters.

Parameter	Unit	Theta		Omega		Shrinkage
		Estimate	RSE (%)	IIV	RSE (%)	(%)
***CL***	L/hr	0.0482 (0.0336, 0.0681)	16.1	0.49 (0.307, 0.738)	16.1	5.3
***V***	L	2.48 (1.77, 2.96)	7	0 (Fixed)	NA	100
***Q***	L/hr	0.0073 (0.000679, 0.0372)	29.7	0 (Fixed)	NA	100
***V***_***2***_	L	4.68 (1.86, 21.9)	24.6	0 (Fixed)	NA	100
***k_tmd_***	hr^-1^	0.217 (Fixed)	0	0 (Fixed)	NA	100
***k***_***ss***_	μmol^-1^	6.21 (3.09, 15.5)	60.1	2.207 (1.498, 2.805)	24.7	29.1
***k_syn_***	μmol^-1^hr^-1^	5.06 × 10^−8^ (Fixed)	0	0 (Fixed)	NA	100
***k_deg_***	hr^-1^	1.63 × 10^−3^ (Fixed)	0	0 (Fixed)	NA	100

*CL*, clearance of rituximab; *V*, volume of distribution for central compartment; *Q*, inter-compartment clearance between central and peripheral compartment; *V*_*2*_, volume of distribution for peripheral compartment; *k*_*tmd*_ *,* complex elimination rate constant; *k*_*ss*_ *,* the steady-state constant; *k*_*syn*_ *,* target production rate constant; *k*_*deg*_, degradation (target elimination) rate constant; RSE, relative standard error; IIV, interindividual variability.

### 3.5 Model evaluation

**FIGURE 1:**
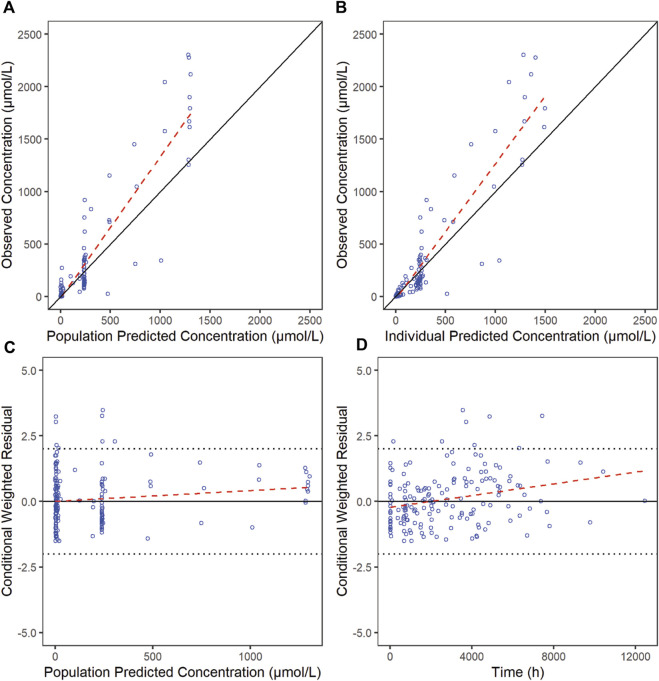
Goodness-of-fit plots for RTX of the final model. The red line is generated by linear method and shows the trend of the data, and the blue circles represent the observed data.

**FIGURE 2:**
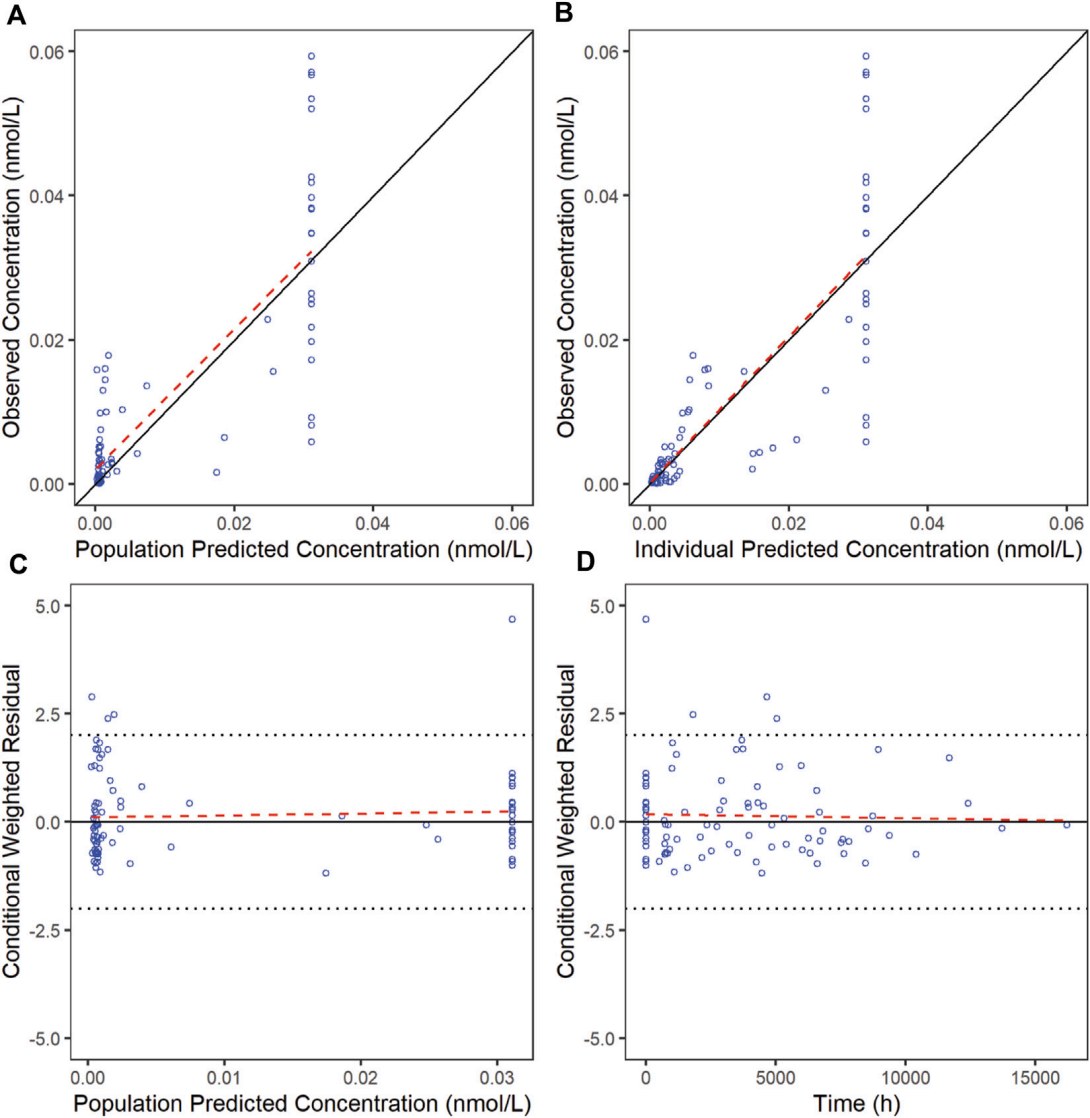
Goodness-of-fit plots for CD20 of the final model. The red line is generated by linear method and shows the trend of the data, and the blue circles represent the observed data.

The goodness-of-fit diagnostic plots of the final model were shown (Figures 1, 2). There was no obvious bias in the model fitting. The VPC and bootstrap results suggested that the model adequately describe the both the RTX concentrations and CD20^+^ B cell counts (Supplementary Figure S2). The general trend and the observed variability were well captured.

### 3.6 Exponentially decrease function of anti-PLA2R titer

Mechanically, anti-PLA2R antibody is excreted by plasma cells, which is in turn transformed from activated B cells that express CD20, and eliminated through catabolism. Thus, once CD20^+^ B cells are depleted by RTX, the plasma B cell count slowly decreases and the production of anti-PLA2R is halted, leading to the subsequent decrease of anti-PLA2R titer. However, we failed to establish the quantitatively relationship between CD20^+^ B cell counts and anti-PLA2R titers. The major reason is that monthly (or even longer) monitor of CD20^+^ B cell counts is incapable to capture the descending process of CD20^+^ B cells as they are rapidly depleted in peripheral blood within 72 h after receiving RTX. Thus, the majority of CD20^+^ B cell count data was obtained either before RTX administration or after depletion (mostly 0/μL) (Supplementary Figure S3A). In addition, the relationship between the recovery of CD20^+^ B cell count and anti-PLA2R titer is controversial. The reemergence of CD20^+^ B cell may be followed either by the increase of anti-PLA2R titer (Supplementary Figure S3B) or not (Supplementary Figures S3C, D). Accordingly, these data are also helpless for modeling.

The anti-PLA2R titers of 36 in all 41 patients were analyzed without the integration of CD20^+^ B cell count. Notably, we found that the anti-PLA2R titer data can be well described using an exponentially decrease function whose *R* ^2^ > 0.8 for all patient except one (*R* ^2^ = 0.67). The function is parameterized with initial anti-PLA2R titer and individual elimination constant *k*_*e,PLA2R*_, which is independent of CD20^+^ B cell counts. The mean *k*_*e,PLA2R*_ is 0.033 ± 0.017, corresponding to the mean half-life 21 days, similar with typical IgG4 antibody half-life. Similar phenomena were also observed in previous studies treated with standard dosage, where the *k*_*e,PLA2R*_ is 0.025 with a *R* ^2^ of 0.98 using titers in the first 3 months (Ruggenenti et al., 2015). We conducted Pearson correlation analysis to explore the physiological factor that influence *k*_*e,PLA2R*_. Unfortunately, no significant relationship (Pearson correlation coefficient > 0.7) was identified.

### 3.7 Simulation of different dosage

**FIGURE 3:**
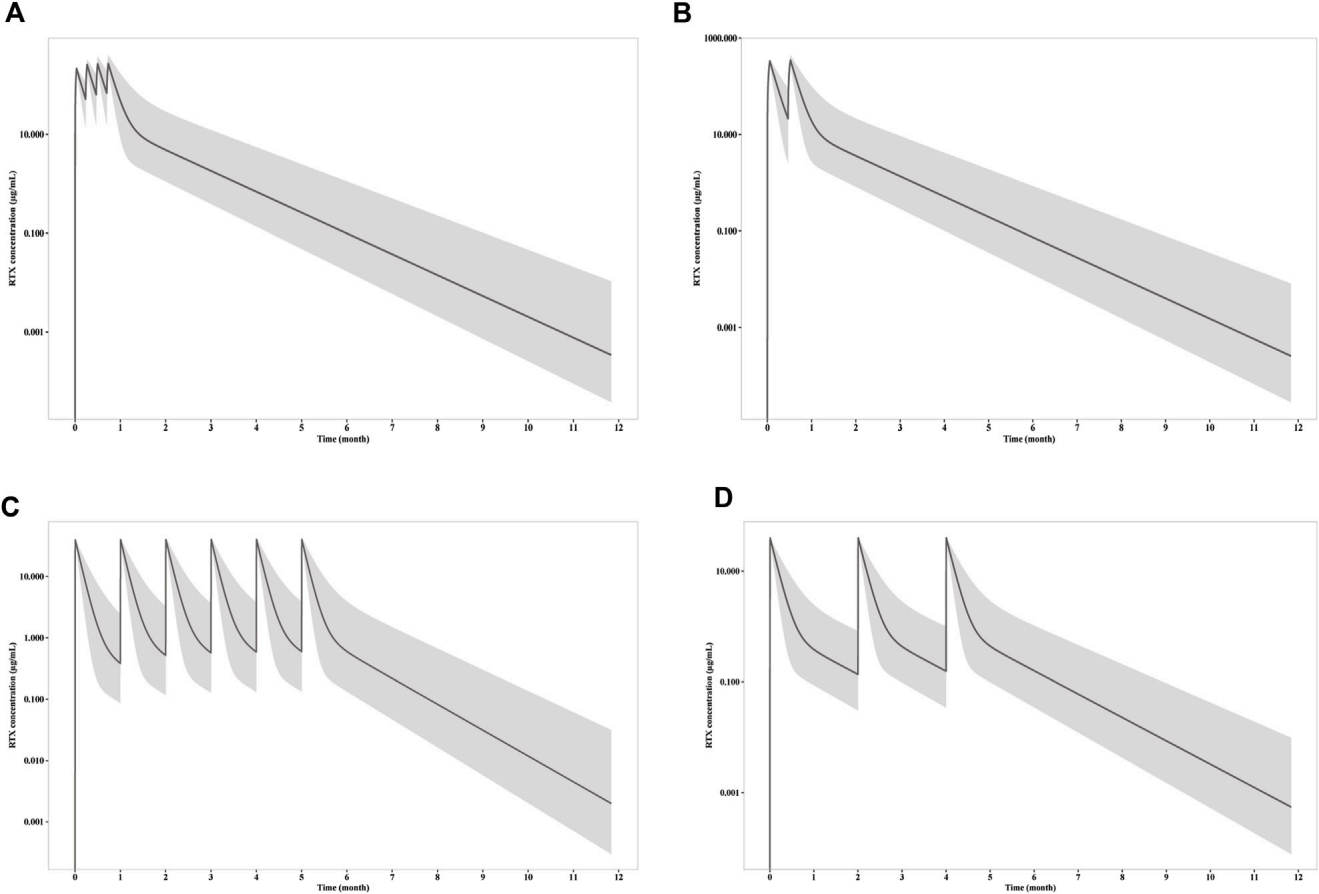
Simulation of RTX concentration after infusion of **(A)** four weekly 375 mg/m^2^ RTX; **(B)** two 1000 mg RTX with two weeks apart; **(C)** six monthly 100 mg RTX; **(D)** three 100 mg RTX with two months apart. The solid line represents the mean RTX concentration versus time, while the shadow represents the 90% prediction intervals.

The simulations were carried out to compare the efficacy of standard dosage 1 (four weekly infusions of 375 mg/m2), standard dosage 2 (two 1000 mg infusion with 2 weeks apart) and mini-dose (six monthly 100 mg). RTX rapidly achieve peak concentration at 215, 332, and 39 μg/mL, whereas the trough concentration collected 1 week after first infusion is 52, 118 and 7.8 μg/mL for standard dosage 1, standard dosage 2 and mini-dose, respectively (Figure 3). The apparent clearance and half-life are 0.54 L/day and 14.7 days, respectively, as calculated by non-compartmental analysis for monthly mini-dose.

**FIGURE 4:**
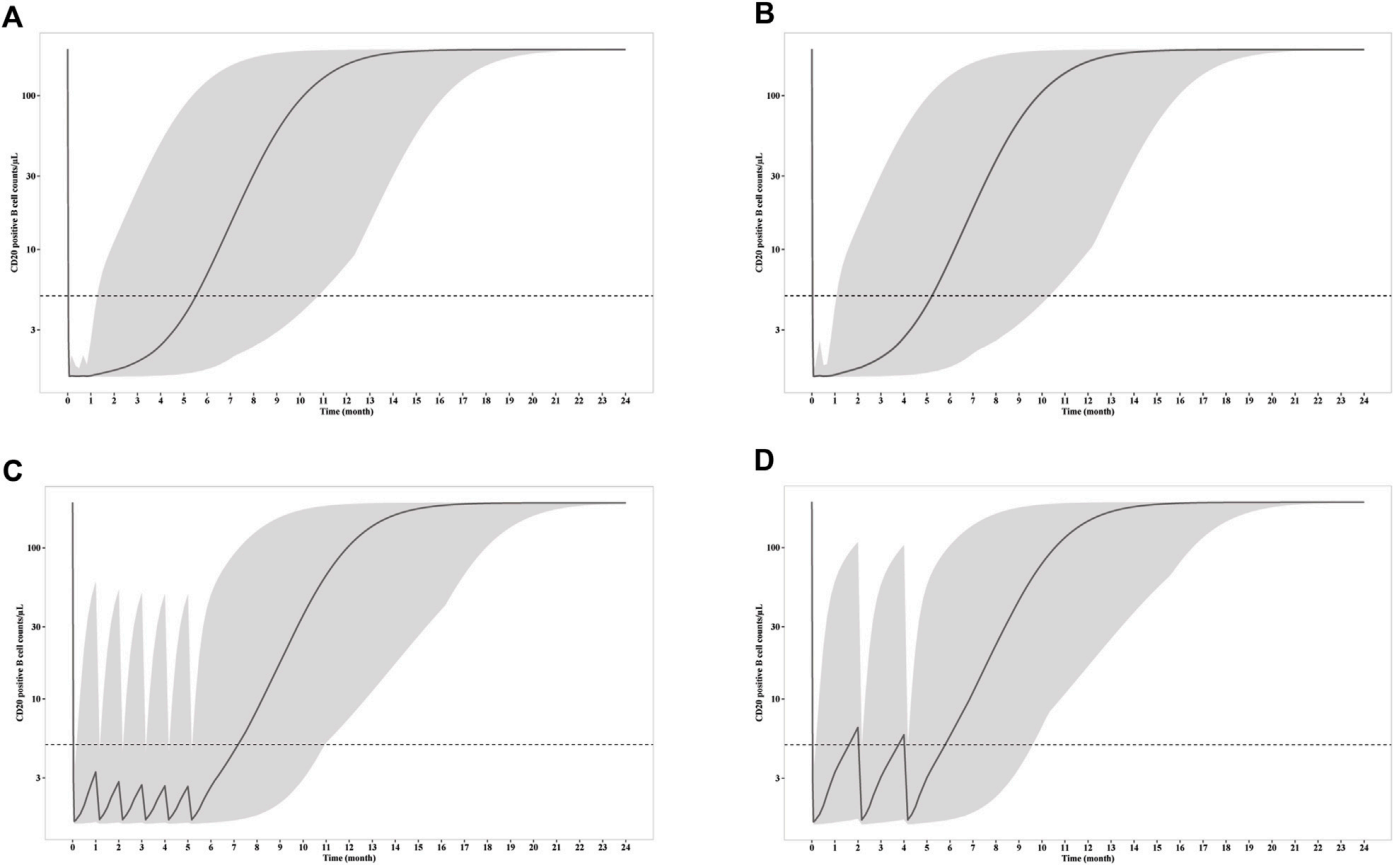
Simulation of CD20^+^ B cell counts after infusion of **(A)** four weekly 375 mg/m^2^ RTX; **(B)** two 1000 mg RTX with two weeks apart; **(C)** six monthly 100 mg RTX; **(D)** three 100 mg RTX with two months apart. The solid line represents the mean CD20^+^ B cell counts versus time, while the shadow represents the 90% prediction intervals. Dashed line indicated CD20^+^ B cell depletion criterion (5/μL).

CD20^+^ B cell depletion was used as a surrogate for clinical efficacy, since the decrease of anti-PLA2R titer occur shortly after the depletion and is independent of B cell recovery as demonstrated above. One the other hand, B cell recovery can be regarded as a marker for safety since it is related with reduced infection incident, which is the predominate adverse effect of RTX in PMN treatment. Though the concentration is much lower for mini-dose, the ability to deplete CD20^+^ B cell is similar (Figure 4). Under all three dosages, the depletions of CD20^+^ B cells occur within 24 h, and slowly recover after last dose. Notably, the duration of depletion is longer for mini-dose than standard dose. CD20^+^ B cell count increases back to more than 5/μL in ∼ 5.5 months for both standard dosages after first infusion, while the depletion can last for more than 7 months for mini-dosage. On the other hand, it takes shorter time for mini-dosage to recover back to normal (90% of baseline CD20^+^ B cell count) than standard dosages (10 months vs 12 months after last dose). Thus, the mini-dose outperforms standard dose with lower total doses (600 mg vs 2000–2400 mg), longer depletion duration (7 months vs 5.5 months) and faster recovery (10 months vs 12 months).

To explore the possibility to further reduce the dosage with larger interval, we simulated the RTX concentration and CD20^+^ B cell count receiving 3 doses of 100 mg every 2 months. Although the RTX concentrations and time for CD20^+^ B cell depletion remain the same with monthly mini-dose in the first week, the duration of CD20^+^ B cell depletion is largely reduced. The median CD20^+^ B cell count increases up to more than 5/μL in less than 2 months. Thus, 100 mg every 2 months dosage is insufficient for long time CD20^+^ B cell depletion and thus should not be recommended for PMN treatment.

## 4 Discussion

PPK model have been established to describe the PK profile of RTX under various disease conditions, including non-Hodgkin’s lymphoma (NHL) (Ternant et al., 2019), diffuse large B cell lymphoma (DLCBL) (Blasco et al., 2009), chronic lymphocytic leukemia (CLL) (Li et al., 2007), follicular lymphoma (FL) (Regazzi et al., 2005), rheumatoid arthritis (RA) (Ng et al., 2005) and anti-neutrophil cytoplasmic autoantibody-associated vasculitis (AAV) (Bensalem et al., 2020). However, PK profile of RTX in PMN patients may be different due to distinct CD20^+^ B cell count (in comparison with B cell lymphoma) or additional elimination of antibody through proteinuria. Indeed, previous study indicated that the clearance of RTX in PMN is ∼2 folds than in follicular lymphoma and autoimmune disorders (Fogueri et al., 2019). Similar PK profile was also observed in our study. The systematic clearance in our study is 0.54 L/h, same as previously reported RTX clearance in PMN and higher than that in B cell lymphoma and other autoimmune disease (Bensalem et al., 2022). The central and peripheral volumes of distribution is 2.48 L and 4.68 L, respectively. The central volume of distribution in our study is slightly lower than that in other disease but 1.9-fold higher than that in previous PMN study. The half-life of a drug means the time it costs for the body to eliminate half of the remaining drug. Shorter half-life is associated with the faster decrease of RTX concentration and may lead to reduced clinical efficacy. The RTX half-life in our study (14.7 days) is comparable to that in previous kidney disease study, such as PMN (11.5 days) (Fogueri et al., 2019), pediatric primary glomerulonephritis (14.6 days) (Zachwieja et al., 2012) and adult minimal change nephrotic syndrome (10–15 days) (Iwabuchi et al., 2018) and is much shorter than that in follicular lymphoma and autoimmune disorders (Regazzi et al., 2005). These evidences indicated that the PK and PD profiles are significantly affected by the urinary loss of RTX, which is consistent with previous studies (Del Vecchio et al., 2021; Teisseyre et al., 2022).

Two-compartment model is frequently used to describe RTX PK in most studies, a simple approach but ignores the nonlinear elimination due to antigen binding. It was recently demonstrated that TMDD model can better capture the PK profile of rituximab (Bensalem et al., 2022). In our study, we constructed four two-compartment models with linear or nonlinear elimination described by time-varying, Michaelis-Menten or TMDD approaches. Consistent with previous study, TMDD model outperform other models with lowest OFV and better fitting. In addition to RTX concentration, TMDD is capable to capture the profile of CD20^+^ B cell count, a PD marker, providing the basis to construct the PK/PD relationship to guide dosage optimization.

CD20^+^ B cell plays a critical role in PMN progress and prognosis. A prospective study was conducted to compare risk and benefit profile of standard four weekly 375 mg/m^2^ RTX dose treatment or B cell-driven treatment (patients receive a second infusion of RTX only if they had CD20^+^ B cell count more than 5/μL). B cell-driven treatments achieved similar reduction of proteinuria with less adverse events and much lower medical costs (Cravedi et al., 2007). A recent study revealed that serum RTX concentration at month-3 (3 month after RTX injection) can well predict the remission rate at month-6 and month-12. Patients with RTX concentrations less than 2 μg/mL at month-3 exhibited weaker CD20^+^ B cell depletion ability, lower clinical remission rate and need longer time to achieve clinical remission (Teisseyre et al., 2021). Collectively, these studies indicated the rational and reliability to use the duration of CD20^+^ B cell depletion as a marker to indicate the therapeutic effect and to balance risk and benefit.

Although the PK/PD model was not established for anti-PLA2R titer due to the sparse sampling of CD20^+^ B cells, an exponentially decrease function was used to predict the reduction of anti-PLA2R. The mean elimination constant k_e,PLA2R_ is 0.033 ± 0.017, corresponding to the mean half-life of 21 days. Six monthly doses of 100 mg RTX infusion is able to maintain the depletion of CD20^+^ B cells for more than 7 months, which is sufficient for the fully elimination of anti-PLA2R antibody (∼10 half-lives). However, a large variability was observed for k_e,PLA2R_ that ranges from 0.01 to 0.079. For some patients, 6 months infusion may be insufficient or unnecessary. Although the factors that influence the individualized elimination constant k_e,PLA2R_ remains elusive, monitoring of anti-PLA2R titer can be used to fit the parameter. After that, the reduction of anti-PLA2R is predictable and dosage can be adjusted accordingly. Therefore, a personalized treatment is feasible, which will be further explored in the upcoming study.

There are still many defects in our study. First, the sample size is still limited, especially when sparse sampling was adopted. Second, we failed to identify any covariates that influence both RTX concentration clearance and anti-PLA2R antibody elimination. Previous studies have identified body weight, sex and body surface area as covariate for clearance under other disease conditions (Wang et al., 2020). For PMN, however, all these covariates have little influence on RTX PK profile. No covariate was reported by other PPK study for RTX usage in PMN as well (Fogueri et al., 2019). More clinical studies will be conducted in the near future to further optimize our model and dosage.

## 5 Conclusion

In this study, we established the quantitative relationship between RTX dosage, PK characteristics, CD20^+^ B cell counts and anti-PLA2R titers by population PK/PD model. The simulation results supported the use of monthly mini-dose for the treatment of PMN patient with comparable efficacy and much lower cost. Our study can also provide insightful view for the clinical development and precision medicine of other anti-CD20 antibodies.

## Data Availability

The original contributions presented in the study are included in the article/Supplementary Materials, further inquiries can be directed to the corresponding authors.
