# Supplementary material for: Dosing optimization of rituximab for primary membranous nephropathy by population pharmacokinetic and pharmacodynamic study
Source: Front Pharmacol. 2024 Mar 26;15:1197651. doi: 10.3389/fphar.2024.1197651 (PMC11002205; doi:10.3389/fphar.2024.1197651)
Supplement: Supplementary file 1 [file DataSheet1.pdf]

Supplementary Material

Dosing optimization of rituximab for primary membranous nephropathy by population pharmacokinetic and pharmacodynamic study

Hao Liang[1,2†], Zhenling Deng[1†], Shu Niu[2†], Weijie Kong[1,2†], Yang Liu[3], Song Wang[1], Haiyan Li[2], Yue Wang[1], Danxia Zheng[1*] and Dongyang Liu[2,4,5*]

[1]Laboratory X, Institute X, Department X, Organization X, City X, State XX (only USA, Canada and Australia), Country

[2]Laboratory X, Institute X, Department X, Organization X, City X, State XX (only USA, Canada and Australia), Country

[1]Department of Nephrology, Peking University Third Hospital, Beijing, China.

[2]Drug Clinical Trial Center, Peking University Third Hospital, Beijing, China.

[3]Department of Pharmacology, College of Pharmacy, Inner Mongolia Medical University, Hohhot, Inner Mongolia, China.

[4]Institute of Medical Innovation, Peking University Third Hospital, Beijing 100191, China

[5]Beijing Key Laboratory of Cardiovascular Receptors Research, Peking University Third Hospital, Beijing 100191, China

[†]These authors contributed equally: Hao Liang, Zhenling Deng, Shu Niu, Weijie Kong

*** Correspondence:**
Dongyang Liu
liudongyang@vip.sina.com

Danxia Zheng
dxzheng@sina.com

1 Supplementary Figures and Tables

1.1 Supplementary Figures

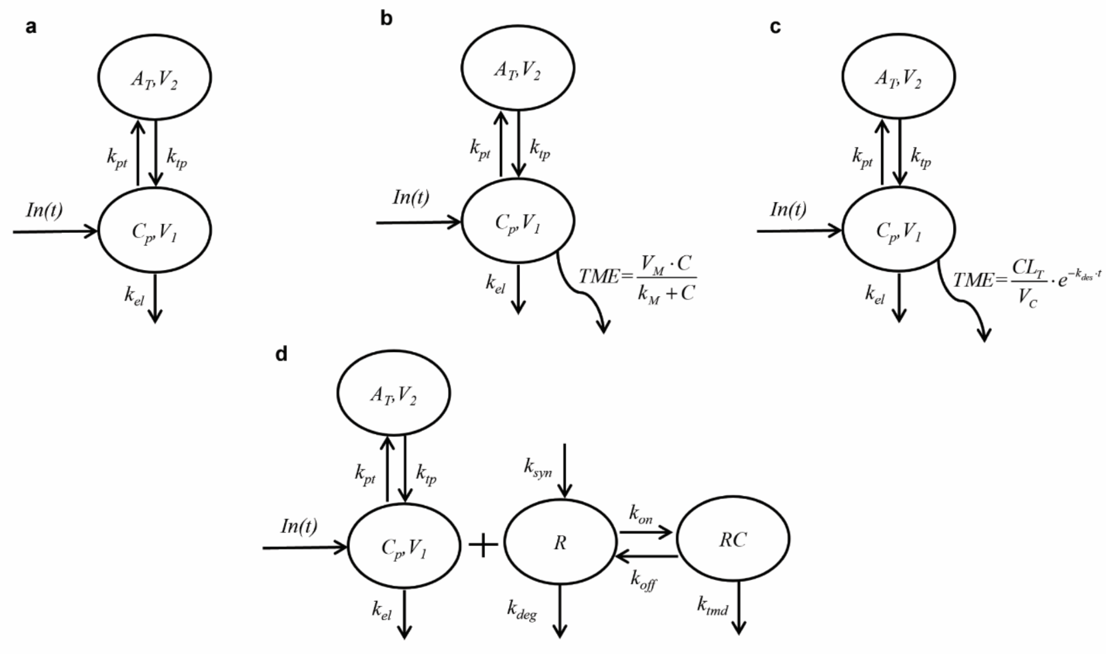

Supplementary Figure 1. Four models tested to describe rituximab pharmacokinetics. (a) Model 1 is defined as 2-compartmental PK model with linear elimination. (b) Model 2 is the model combined model 1 with Michaelis–Menten elimination. (c) Model 3 is described as model 1 with a time-dependent elimination rate. (d) Model 4 is target-mediated drug disposition model.

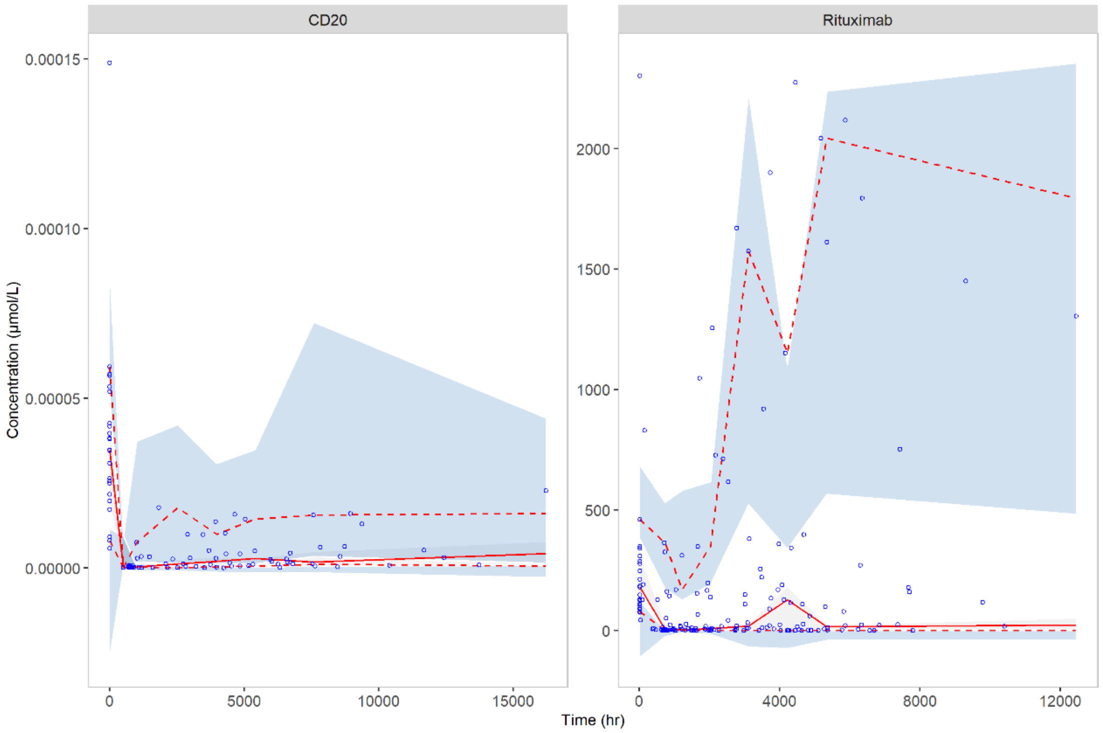

Supplementary Figure 2. Visual predictive checks for the final population PK model with CD20 (left) and RTX (right). The blue circles represent the observed data. The lower and upper dashed lines represent the 5th and 95th percentiles for the observed data. The solid line represents the 50th percentile for the observed data. The shaded areas represent the 95% confidence intervals for the 5th, 50th, and 9^{5th} percentiles of the simulated data.

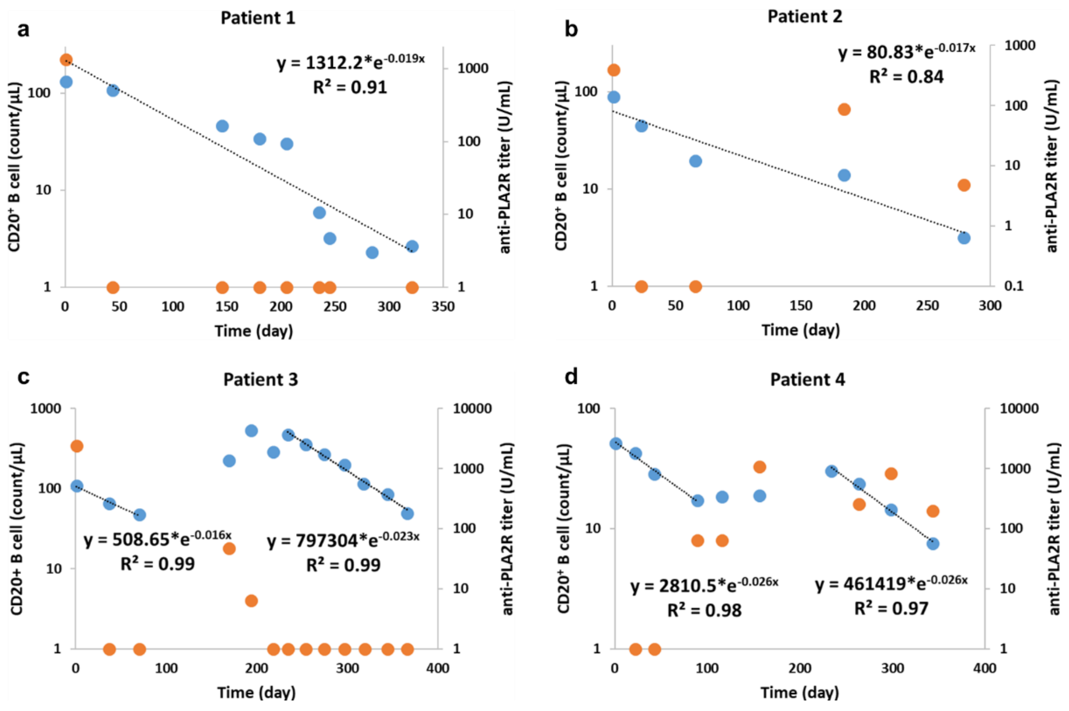

Supplementary Figure 3. The relationship between $CD20^+$ B cell counts and anti-PLA2R titers of representative patients. $CD20^+$ B cell count and anti-PLA2R titer data is displayed as blue and orange dots, respectively. Trendline of exponentially decrease function is shown as black dotted lines.

1.2 Supplementary Tables

Supplementary Table 2. Comparison of different PPK models

Model	Model description	Object function value (OFV)	Key parameters	Residual error model
1	two-compartmental model with linear elimination	1293.4	k_{el}, k_{tp}, k_{pt}, V_1, V_2	proportion
2	two-compartmental model with Michaelis-Menten elimination	1293.5	k_{el}, k_{tp}, k_{pt}, V_1, V_2, V_M, K_M	proportion
3	two-compartmental model with time-varying elimination	1308.8	k_{el}, k_{tp}, k_{pt}, V_1, V_2, CL_T, k_{des}	proportion
4	TMDD model with QSS approximation	-908.3	k_{el}, k_{tp}, k_{pt}, V_1, V_2, k_{syn}, k_{deg}, k_{tmd}, k_{ss}	proportion

k_{el}, first-order elimination rate constant; k_{tp} and k_{pt}, distribution parameters from central to peripheral compartment and from peripheral to central compartment, respectively; V_1 and V_2, central and peripheral volume of distribution; V_M, maximum rate; K_M, Michaelis constant; CL_T and k_{des}, initial target-mediated clearance and its time-decrease rate constant, respectively; k_{syn}, target production rate constant; k_{deg}, degeneration (elimination of the target) rate constant; k_{tmd}, complex elimination rate constant; k_{ss}, the steady-state constant.
